# NO supplementation for transfusion medicine and cardiovascular applications

**DOI:** 10.4155/fso.15.51

**Published:** 2015-08-01

**Authors:** Pedro Cabrales, Daniel Ortiz, Joel M Friedman

**Affiliations:** 1 Department of Bioengineering, University of California, San Diego, La Jolla, CA 92093, USA; 2 Department of Physiology & Biophysics, Albert Einstein College of Medicine, 1300 Morris Park Ave, Bronx, NY 10461, USA

**Keywords:** blood transfusion, hemorrhagic shock, no releasing nanoparticles, red blood cells

## Abstract

Blood transfusions are used to treat reduced O_2_-carrying capacity consequent to anemia. In many cases anemia is caused by a major blood loss, which also creates a state of hypovolemia. Whereas O_2_ transport capacity is restored by increasing levels of circulating Hb, transfusion does not resolve the hypoperfusion, the hypoxia and the inflammatory cascades initiated during the anemia and hypovolemia. This explains why blood transfusion is not always an effective treatment and why transfusion of stored blood has been associated with increased morbidity and mortality, especially in patient populations receiving multiple transfusions. Epidemiologic data indicate that adverse events after transfusion are relatively common, having a great impact on the patients outcome and on the costs of public health. In this chapter, we explain why classical transfusion strategies target the reversal of hypoxia only, but do not address the inflammatory cascades initiated during anemic states and the importance of the flow and vascular endothelium interactions. We also establish the relation between red blood cells storage lesions, limited NO bioavailability and transfusion-associated adverse events. Lastly, we explain the potential use of long-lived sources of bioactive NO to reverse the hypoxic inflammatory cascades, promote a sustained increase in tissue perfusion and thereby allow transfusions to achieve their intended goal. The underlying premise is that adverse effects associated with transfusions are intimately linked to vascular dysfunction. Understanding of these mechanisms would lead to novel transfusion medicine strategies to preserve red cell function and to correct for functional changes induced by hemoglobinopathies that affect cell structure and function.

**Figure 1.**
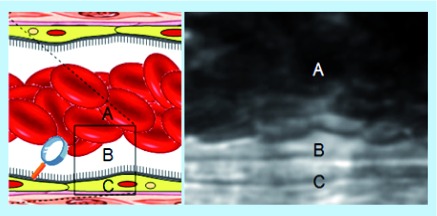
**Blood flow and endothelium interactions in the microcirculation.** Shows schematic representation of the CFL and the RBC core and how it is visualized *in vivo* using intravital microscopy. **(A)** RBC core, **(B)** CFL and **(C)** endothelial lining.

**Figure 2.**
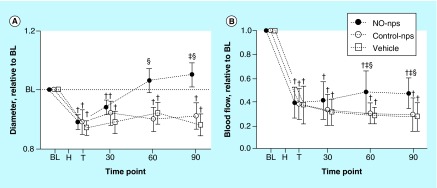
**Microvascular diameter and blood flow during hemorrhagic shock, with and without nitric oxide supplementation.** The infusion of NOnps promote an increase in blood flow, mainly determined by an increase in vascular diameter. This increase in blood flow promotes an increase in O_2_ delivery and thus reduces tissue damage. NOnp: Nitric oxide-releasing nanoparticle.

**Figure 3.**
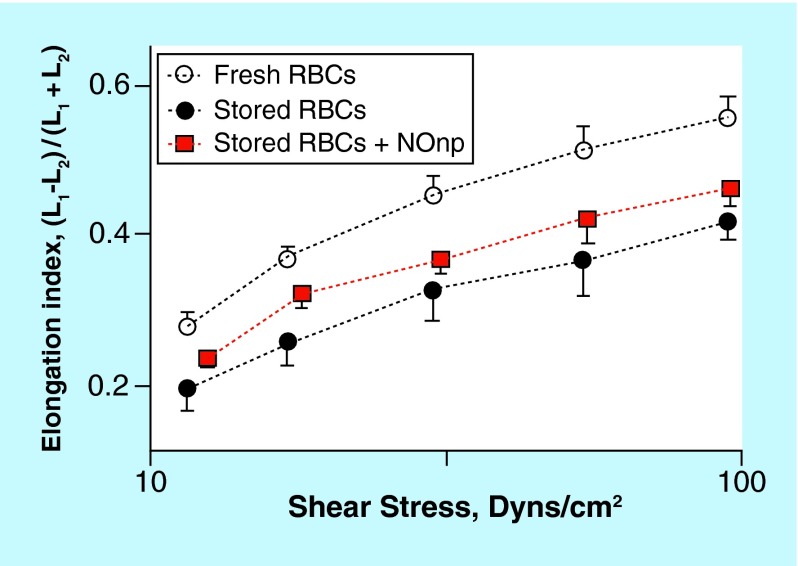
**Red blood cell mechanical changes during storage for 4 weeks with nitric oxide supplementation based on nitric oxide-releasing nanoparticles.** Elongations index as a function of shear stress after 4 weeks of storage. NO-np: Nitric oxide-releasing nanoparticle.

**Figure 4.**
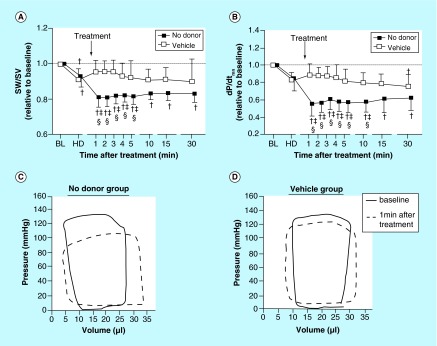
**Effect of nitric oxide supplementation on ventricular function.** The hamster closed chest method was used to simultaneously record pressure and volume of the left ventricle using a conductance catheter, during normovolemic anemia with or without NO supplementation. **(A)** SW/SV and **(B)** dP/dt_min_ were decreased after the NO supplementation. **(C & D)** show examples of the effect of supplementing NO on the ventricular pressure–volume relationship. SW/SV: Stroke work per stoke volume.

Nearly 5 million Americans receive blood transfusions each year. According to the 2011 National Blood Collection and Utilization Survey Report, 14 million units of blood were transfused that year in the USA, and severe transfusion-related adverse reactions were reported in 9.5% of the cases for allogeneic and 1.8% for autologous transfusions [1]. Transfusion-related adverse events are among the costliest contributors to healthcare cost, even without including future illness, lost wages and impact on quality of life.

Blood transfusions are administered after major blood losses or in case of severe anemia. During normal conditions, the systemic circulation and the circulation at the microvascular level are in a state of homeostatic equilibrium. The interaction of blood flow with the vascular endothelium stimulates the production of endogenous NO, and red blood cell (RBC) membrane integrity provides a barrier to Hb–NO reactions. Additionally, adequate blood flow in arterioles and capillary beds assures the meeting of the metabolic demands of every tissue. However, after a major insult such as the loss of significant volumes of blood, or during cardiovascular surgeries, such homeostatic equilibrium is impaired and a proinflammatory condition is initiated, which at the microvascular level is characterized by a marked decrease in local blood flow and tissue oxygenation. In many cases, the intervention (blood transfusion) acts as a second insult mainly because: changes in the flow endothelium interactions, increased NO scavenging by free Hb when RBC sensitive to hemolysis are transfused and the reperfusion/reoxygenation state triggered by the restored O_2_-carrying capacity and overall blood flow. The severity of this secondary insult depends on the quality of the infused blood derivatives and the severity and duration of the ischemic or shock period. The presence of RBC damage or storage lesions causes disturbance in the cardiovascular homeostasis at different levels, many of which are closely related to the availability and function of NO. Restoration of NO production, availability and function could be a key point in the determination of a transfusion success.

## Limited NO before & after blood transfusion

The reduced NO availability during an ischemic situation is the result of decreased endothelial shear stress (due to low blood pressure, low cardiac output, reduced circulating volume and low blood viscosity) [2], uncoupled eNOS reactions resulting in ROS production instead of NO [3], and hypoxia, as O_2_ is a substrate for NO production [4]. Restoration of NO bioavailability prior to a transfusion restores cardiovascular homeostasis and allows transfusion to achieve its intended goal. We have proposed that NO levels can be restored using exogenous NO and bioactive NO forms (nitrite and S-nitrosothiols) [5].

Treatment of severe bleeding after hemorrhage heavily focuses on restoration of O_2_-carrying capacity and intravascular volume. There are clear indications that the restoration of NO bioavailability, reduced during hemorrhage and hypovolemia, reduces inflammatory and ischemic effects [6,7]. Previous studies have shown that the most effective transfusion and fluid resuscitation strategies are those that can best restore the microvascular function. Moreover, fluids that promote an increase in the endothelium shear stress (and thus NO production) have a more beneficial impact in the tissue viability [8]. Resolving vascular malfunction induced by hemorrhagic shock through NO supplementation might prevent inflammation and development of a multiple organ failure [6].

## Blood flow & vascular endothelium interactions: the role of storage lesions

Red cell motion in small blood vessels is an important determinant of blood rheology and flow endothelium interactions. Under normal conditions, RBC are continuously directed toward the center of the vessels by shear forces which create a RBC core and a cell-free layer (CFL) [9]. The CFL acts a hydrodynamic layer that reduces the resistance to flow. The CFL thickness and dynamics determines the rate of O_2_ delivery and NO scavenging by Hb in the RBCs. The CFL thickness is primarily determined by hematocrit and cell hydrodynamic migration away from the wall. Using high temporal and spatial resolution intravital microscopy, we have characterized the dynamics of the CFL after the transfusion of either stored or fresh RBCs, a sample image presenting the phenomena is shown in Figure 1. RBC mechanical properties determine cell deformations under shear forces, cell hydrodynamic responses and thus CFL thickness and local vascular wall shear stress. The RBC storage affects cell mechanical properties and blood rheology compromising blood hemodynamics, O_2_ delivery and the interaction between flowing blood and the vasculature [10]. It has been proposed that limited NO bioavailability contributes to adverse cardiovascular events, including those associated with transfusing stored RBCs [11]. Hemolysis from stored and damaged cells, results in acellular Hb and Hb-containing microparticles in the circulation, scavenging NO and triggering a proinflammatory cascade [12–14]. Recent studies have suggested that the restoration of normal levels of NO bioactive species in the blood to be transfused can be help increase the efficacy of blood transfusions [15], suggesting additional storage lesions associated with NO availability.

## NO depletion after transfusion

From the moment they are withdrawn, erythrocytes begin to exhibit structural and biochemical changes that increase upon manipulation, storage and reinfusion, thereby increasing intravascular hemolysis post-transfusion. Our laboratories have shown that even low levels of acellular Hb disrupt NO levels, via NO dioxygenation reactions [16]. At the smooth muscle level, this process limits the activation of NO receptor, soluble guanylate cyclase, resulting in decreased cyclic guanosine monophosphate causing vasoconstriction.

## Reactive oxygen species & transfusion

Transfusion restores O_2_-carrying capacity, and produces a reperfusion/reoxygenation injury as perfusion is recovered. Protection against ischemia-reperfusion has motivated intense study. Ischemia-reperfusion-reoxygenation injury is strongly linked to oxidative stress and the formation of ROS. ROS react with cellular macromolecules, such as proteins, lipids and nucleic acids, affecting cellular machinery. In vascular endothelial cells, ROS consume NO, induce vascular inflammation and endothelial dysfunction [17]. Intravascular restoration of vascular NO levels will sustain vascular endothelial function, break oxidative damage and interfere with ROS chain reactions.

## Limitations of available NO delivering platforms

NO is a lipophilic, diatomic, free radical that is stable and soluble in aqueous solutions when compared with other radical species; however, intravascular NO supplementation is a challenge. NO is rapidly scavenged by Hb, resulting in a very short intravascular half life. Currently, the clinical therapeutic potential of NO has only been exploited via inhaled NO. While this approach is inconvenient and costly, inhaled NO is the only approved NO treatment for specific pathologies such as acute pulmonary hypertension [18]. Alternatives for intravascular NO therapy include formulations based on compounds containing either NO or an NO precursor; however they have the disadvantage of not being amenable to controlled and sustained delivery [19,20]. NONOates (diazeniumdiolates) comprise the largest family of inorganic NO donors. They are complexes of NO with nucleophiles, their decomposition rate depends on pH, temperature and the nature of the nucleophile [21]. After establishing their ability to generate NO in *in vitro* experiments, some of them (Diethylamine [DETA/NO] and spermine [SPER/NO]) have been used in experimental animal models to modulate vascular tone, thrombosis and neointimal hyperplasia [22–24]. Other NONOates have been used successfully in animal models to protect against nephrotoxicity and hepatotoxicity [25,26]. However, NONOates has also been shown that to convert to the highly cancerous agent N-nitrosopyrrolidine [27]. Thus, even after their successful application in experimental models the difficulty to establish a safety profile for the different available compounds has slowed its translation to the clinical use [27].

## Sustained release of NO, nitrite & S-nitrosothiols

NO, nitrite and S-nitrosothiols have significant therapeutic potential in transfusion medicine that has not yet been understood. Inhaled gaseous NO prevented pulmonary and systemic hypertension induced by transfusion, although the observed beneficial effects of inhaled NO were most limited to pulmonary hemodynamics. Other approaches to resolve NO dysregulation induced by transfusion included nitroglycerin supplementation, which transiently decreased the vasoconstriction. Nitroglycerine is an organic nitrate, releases NO from a three-electron reduction process. Nitroglycerine bioactivation involves specific enzymes, limiting nitroglycerin effectiveness as these enzymes are depleted, an effect called nitrate tolerance.

## Intravascular delivery of NO based on nanoparticles

Ongoing research has revealed the therapeutic potential for NO in treating infection and in modulating vasoactivity, angiogenesis and wound healing. Harnessing this potential poses a challenge, especially the development of a convenient or cost-effective approach. The objective, then, is to develop therapeutic strategies that sequester the NO functionality into a chemically stable carrier that can be directed to the deficient site to carry out its functions without systemic consequences. Early approaches to delivering NO using nanotechnology included gold nanoparticles (1–5 nm) covered with amino groups reacted with NO [28]. Gold nanoparticles were synthesized and functionalized with bromo-terminated alkanethiols and then reacted with amine groups [28]. Lastly, the amine-functionalized gold nanoparticles were pressurized with NO and stirred to facilitate the synthesis of NO donors and prevent aggregation. Although, gold nanoparticles are a flexible technology to deliver NO, the total amount transported is limited.

## Hybrid sol-gel NO-releasing nanoparticles

Currently a biocompatible platform that utilizes a hydrogel precursor in conjunction with chitosan and polyethylene glycol to form a fine powder of NO-releasing nanoparticles its being developed [19,20]. The redox chemistry of a glucose solid matrix is used as the basis to generate a powder formulation, capable of delivering NO in a sustained fashion. Briefly, a hydrogel-glass composite is synthesized using a mixture of tetramethylorthosilicate, polyethylene glycol, chitosan, glucose and sodium nitrite in 0.5 M sodium phosphate buffer (pH 7). Nitrite is reduced to NO within the matrix because of the glass properties of the composite affecting redox reactions initiated with thermally generated electrons from glucose. After the redox reaction, the ingredients are dried using a lyophilizer, resulting in a fine powder composed of nanoparticles containing NO. Once exposed to an aqueous environment, the hydrogel properties of the composite allowed for the opening of water channels inside the particles, facilitating the release of trapped NO over extended time periods.

Systemically, a blood transfusion should recover blood pressure (BP), cardiac output (CO) and vascular resistance (VR) to baseline levels after resuscitation from hemorrhagic shock. Although when systemic parameters appeared to be restored after fluid resuscitation from hemorrhagic shock, microvascular blood flow after resusciation depends on the physical properties of the fluid during resuscitation. Experimental evidence has shown that fresh blood recovers the microcirculation better than stored blood, and that high viscosity plasma expander are more beneficial than low viscosity plasma expanders. The supplementation of NO serves to compensate for the loss in shear stress and promotes the relaxation of the microscopic blood vessels, which also contributes to an increase in the local blood flow. Our NO-releasing nanoparticles (NOnps) have shown a high efficacy in relaxing vascular tone compared with NONOates [29]. About 30-times less NO is required from NOnps compared with NONOates to produce similar dilation when applied intravascularly. The effects of NOnps were sustained longer compared with NONOates, which only lasted minutes [29]. The higher doses of NO required by NONOate significantly increases metHb levels, decreasing O_2_ carrying capacity [29]. Figure 2, shows the effect of infusing NOnps during resuscitation from hemorrhagic shock as studied using a hamster window chamber model [30]. In this model, microvascular arteriolar and venular diameters and blood flow were measured at baseline conditions, after a severe hemorrhage (50% of the blood volume, H) after treatment (T, NOnps, Control nps or vehicle) and up to 90 min after the treatment. The experiment evidenced the ability of the NOnps, to maintain a relaxed vascular tone (vasodilation) and thus an improved blood flow, even in conditions of hemorrhagic shock with no fluid treatment.

## Role of low NO supplementation during blood storage

Biochemical and mechanical red cell changes of stored red cells during storage were studied during NO supplementation with NO-nps (in a molar ratio of 1 NO per 1000 Hb every 48 h). NO-nps dose was calculated based on a NO-releasing capacity for the NO-nps of 0.3 μmol of NO per mg of NO-nps (4 h at 37°C and pH 7.4) [31]. NO-nps were suspended in a small volume of PBS buffer and mixed into blood storage bag. Figure 3 shows RBC mechanical changes during storage with NO supplementation form NOnps. RBCs from humans, hamsters and rats were leukodepleted and stored in CPDA-1. In all species, NOnps delayed or prevented chemical changes (2,3 DPG depletion, K^+^ leakage, drop pH and hemolysis). NO prevented the changes in RBC deformability due to storage for 2 and 4 weeks, especially at low shear rates. MetHb levels were below 5% in all the cells receiving NO supplementation. ATP production and glucose consumption of cells stored with NO supplementation were greater than without NO, suggesting changes in RBC metabolic activity.

## Role of NO supplementation on left ventricular function

Just as the preservation of the microvascular function, the success of a fluid therapy for the treatment of hemorrhagic shock is greatly determined by the maintenance of an adequate cardiac function. In particular, the left ventricle is subjected to great stress due to the increase in vascular resistance (afterload) and the limited blood supply during the shock period. We have investigated the effect of using exogenous NO supplementation in the form of NONOates, on the cardiac function during a normovolemic anemic state. Using a closed chest method, we analyzed the changes on ventricular pressure and volume overtime after hemodilution with Dextran 70 (Dex70) and after the treatment with an NO donor. The cardiac function was characterized in terms of clinically relevant hemodynamic parameters [32]. Our results indicate that stroke work per stoke volume and the minimum rate of pressure change (dP/dt_min_) were significantly lower in NO-treated animals. Importantly we found the NO effects to be dose dependent and short lasting. These parameters are shown in Figure 4A & B. Additionally, Figure 4C & D shows the relationship between ventricular pressure and volume (pressure–volume [PV] loops). In the NO donor group the loop shows a decrease in the maximum ventricular pressure and an increase in the final ventricular volume (increased preload). Those two changes suggest a decrease in the systemic vascular resistance.

Our results suggest that NO donors could be used as enhancers of ventricular function during critical steps of a resuscitation procedure. However, several studies have shown that NO could have bimodal effects over the cardiac function: either cardioprotective or cardiodepressive, depending on the dosage. Animals treated with a high dose of NONOates in our study did not survive the protocol, whereas animals treated with low dosage did not show any significant response. Further experimental evidence is needed to establish the exact role of NO supplementation of cardiac function both, at normal and pathological conditions.

## Conclusion

Ever since the discovery of the role of NO in the cardiovascular physiology, many approaches have been taken to use it as a therapeutic agent. However, the difficulties in achieving stable and sustained concentrations in the intravascular media have limited such development. Here, we presented some current ideas and developments on how NO supplementation could be used to improve the efficacy of blood transfusions at the microvascular level. Importantly the fact that NO supplementation could increase the shelf life and quality of banked blood would have an important impact on public health. Also, some of the work performed by our group and others, aim to understand how to effectively supplement a safe dosage of NO, to produce beneficial changes in the ventricular function. Any development carried out in these areas is of great clinical relevance.

**Executive summary**

Blood transfusion is one of the most common medical therapies; however, a growing body of literature has demonstrated an association between adverse clinical outcomes and blood transfusions.
Chemical and morphological changes in red cell occur during blood storage.
Cell free hemoglobin released from transfused blood increases inflammation and nitric oxide scavenging.
NO functions as the endothelial-derived relaxing factor, and studies have shown that blood transfusion is associated with a loss of nitric oxide signaling.
NO reduced mechanical changes in red blood cells during storage.
